# 

**Published:** 1892-10

**Authors:** 


USES OF WATER IN MODERN MEDICINE, Vol. 1, Leisure Library Series; by Simon Baruch, M. D. Paper back. Pp. 115. Price 25 cents. Geo. S. Davis, publisher, Detroit, Mich. 1892.

This little book does not attempt to bring water forth as a "cure all," but endeavors to give it its legitimate place in therapeutics, an object indeed worthy the best efforts of the able author and for which the American profession ought to exhibit gratitude in a substantial manner. B.

